# Hospital volume and efficiency in transcarotid transcatheter aortic valve replacement: Insights from a national database of 10,000+ cases

**DOI:** 10.1016/j.xjse.2026.100123

**Published:** 2026-05-04

**Authors:** Gloria Zacharias, Connor Moynihan, Daohai Yu, Xiaoning Lu, Karan Patel, William Moser, Suyog Mokashi

**Affiliations:** a Lewis Katz School of Medicine at Temple University, Philadelphia, Pa; b Department of Biomedical Education and Data Science, Lewis Katz School of Medicine at Temple University, Philadelphia, Pa; c Division of Cardiovascular Surgery, Temple University Hospital, Philadelphia, Pa

**Keywords:** transcatheter aortic valve replacement, transcarotid TAVR, hospital volume, clinical outcomes, length of stay, ICU utilization, resource optimization

## Abstract

**Background:**

When transfemoral transcatheter aortic valve replacement (TAVR) is unsuitable, transcarotid TAVR offers a safe alternative. The relationship between hospital procedural volume and clinical outcomes remains unclear, and solidifying this relationship is key to establishing benchmarks and guiding high-quality care.

**Methods:**

We retrospectively analyzed US academic centers in the Vizient Clinical Database, a national repository of tertiary and quaternary hospital data. All centers performing transcarotid TAVR in 2022 to 2024 were included. Patient demographics and outcomes were compared using the Kruskal-Wallis and χ^2^ tests. Spearman rank correlation was used to assess hospital volume and outcome associations.

**Results:**

Between 2022 and 2024, 118 academic hospitals performed 10,394 transcarotid TAVR procedures. Institutions were stratified by 3-year cumulative procedural volume: Q1, 15 to 47 cases (n = 87); Q2, 48 to 77 (n = 87); Q3, 78 to 111 (n = 90); and Q4, 112 to 312 (n = 90). High-volume centers treated older patients (71 years vs 69 years), a higher proportion of white patients (85.4% vs 64.5%), more Medicare beneficiaries (72.4% vs 62.8%), and patients with lower case complexity (Case Mix Index, 2.6 vs 3.5) (*P* < .0001 for all). Observed hospital length of stay (LOS) decreased across quartiles (7.5 to 4.9 days), and intensive care unit admission declined (70.7% to 42.5%) (all *P* < .0001). Observed mortality and mortality index did not differ significantly (all *P* > .05). Spearman correlation analysis showed weak associations between higher volume and shorter LOS or lower case complexity, with very weak and inconsistent correlations for mortality.

**Conclusions:**

Higher hospital transcarotid TAVR volume is associated with greater resource efficiency, likely attributable in part to lower patient complexity. Mortality and complication outcomes were roughly comparable between transcarotid and transfemoral TAVR, suggesting comparable short-term safety across institutions.

## Graphical Abstract

Higher hospital procedural volume for transcarotid TAVR was associated with greater resource efficiency, reflected by shorter length of stay and reduced ICU utilization, while mortality remained comparable across volume quartiles. These findings inform performance benchmarking focused on efficiency and care processes rather than on procedural volume alone.

**Figure:**
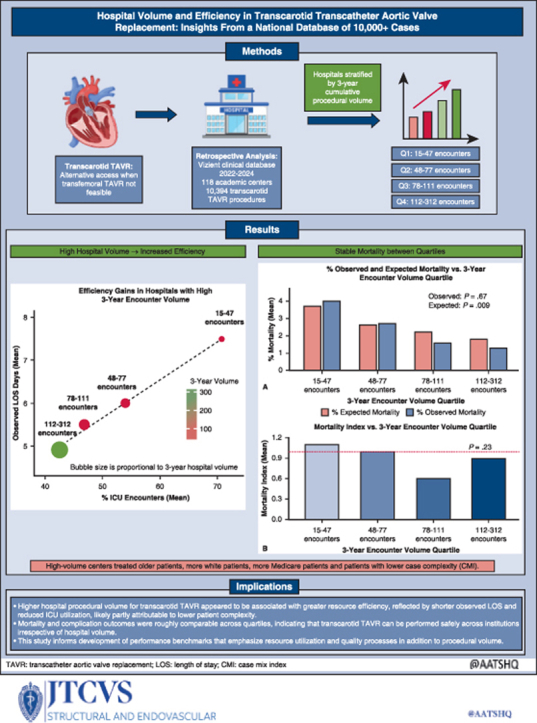

**Figure:**
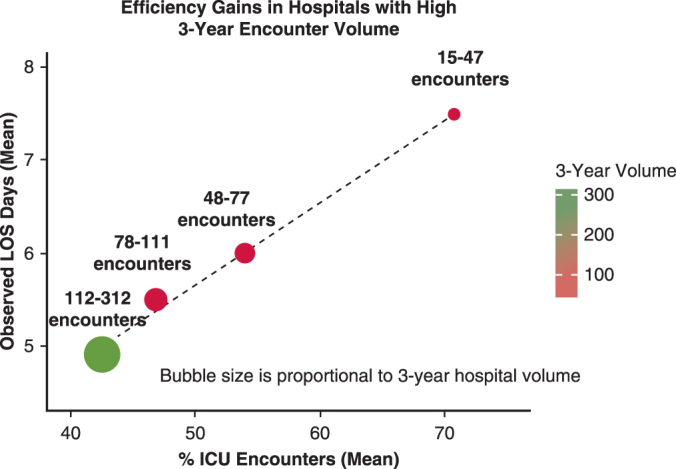
Higher-volume hospitals achieve lower LOS and % ICU use, highlighting efficiency gains.

**Central Message**

Greater experience with transcarotid TAVR, reflected by higher case volume, drives gains in efficiency without compromising mortality, supporting discussions on performance benchmarks.

**Perspective**

As transcarotid TAVR use expands for patients ineligible for transfemoral access, hospital experience may influence efficiency and resource utilization. Our analysis demonstrates that higher-volume centers achieve shorter length of stay and lower ICU use without affecting early outcomes, supporting the safe expansion of this approach while optimizing care delivery and resource allocation.

See Commentary on page XXX.

Transcatheter aortic valve replacement (TAVR) has evolved substantially since its 2011 approval for high-risk patients. Prior work has demonstrated an inverse association between institutional TAVR procedural volume and short-term clinical outcomes, particularly early mortality.^1^ Large registry analyses and systematic reviews report lower 30-day mortality and fewer major complications at higher-volume centers, even after risk adjustment and exclusion of early program learning periods.^1^ More recent analyses incorporating both transcatheter and surgical aortic valve replacement have shown that higher total institutional aortic valve replacement volume is associated with lower in-hospital mortality, emphasizing the importance of system-level experience and multidisciplinary heart team performance.^2^

While transfemoral access remains preferred, patients require an alternative access strategy when iliofemoral anatomy is unfavorable. Nonfemoral access accounted for approximately 4% of TAVR procedures performed after 2020.^3^ Transcarotid access accounted for 2.1% of all TAVR cases in 2023, becoming the most frequently used alternative access route,^4^ although traditionally axillary/subclavian access was used more frequently.^5^ In a large propensity-matched analysis, transcarotid TAVR was associated with lower short-term mortality, reduced atrial fibrillation, and shorter intensive care unit (ICU) and hospital length of stay (LOS) compared with transthoracic access.^6^ Compared with transaxillary or subclavian access, transcarotid TAVR demonstrated similar mortality but a lower stroke rate in a propensity-matched registry analysis.^7^ Comparisons with transfemoral access further suggest that transcarotid TAVR can achieve similar in-hospital and 1-year outcomes despite a higher baseline disease burden among patients selected for alternative access.^8^

Most prior analyses focused on transfemoral cohorts or combine multiple alternative access routes, limiting insight into access-specific patterns. Transcarotid TAVR historically has accounted for only a small proportion of cases,^6^ restricting evaluation of center-level volume effects. Transcarotid TAVR differs from transfemoral TAVR in technical execution and patient selection, which may influence how procedural volume relates to outcomes. Accordingly, the primary objective of this study was to examine the association between hospital transcarotid TAVR procedural volume and clinical outcomes using a national database capturing more than 10,000 cases. Secondary objectives included the assessment of efficiency metrics across hospital volume strata and evaluation of whether established TAVR volume–outcome principles extend to transcarotid access.

## Methods

### Study Design and Data Source

We conducted this retrospective cohort study using the Vizient Clinical Database, which contains deidentified administrative and clinical data from more than 130 US academic medical centers. The database captures patient demographics, diagnoses, procedures, clinical outcomes, resource utilization, and quality metrics. The dataset is provided in an aggregated format for analytic use; therefore, patient characteristics and outcomes are available as summary measures within each hospital-volume category and hospital-year observation rather than as a fully linkable individual patient record with covariate-level detail. The study cohort included 10,402 transcarotid TAVR encounters performed at 118 US academic medical centers between January 1, 2022, and December 31, 2024, representing 354 hospital-year observations. All academic TAVR centers in the Vizient database were included, having performed at least 1 transcarotid TAVR during the study period. Transcarotid TAVR procedures were identified using International Classification of Diseases, Tenth Revision procedure codes and institutional procedure classifications. Institutions were stratified into quartiles based on aggregated 3-year transcarotid TAVR procedural volume, calculated at the hospital level and analyzed at the hospital-year level. Quartile definitions were as follows: quartile 1 (Q1), 15 to 47 cases (n = 87 hospital-years); Q2, 48 to 77 cases (n = 87 hospital-years); Q3, 78 to 111 cases (n = 90 hospital-years); and Q4, 112 to 312 cases (n = 90 hospital-years).

### Outcomes

Primary outcomes included in-hospital mortality (observed mortality, expected mortality, and mortality index) and efficiency metrics (observed length of stay [LOS], expected LOS, and LOS index). Secondary outcomes included percent intensive care unit (ICU) utilization, mean ICU days, and procedural complications. Patient complexity was assessed using the hospital-level Case Mix Index (CMI).

### Statistical Analysis

Descriptive statistics were calculated for all variables. Patient-level analyses included 10,402 encounters, while hospital-level analyses included 354 hospital-year observations. Continuous variables are summarized as mean with standard deviation, and categorical variables are reported as frequency and percentage. Comparisons across volume quartiles were performed using the Kruskal-Wallis test for continuous variables and chi-square tests for categorical variables. Associations between hospital procedural volume (modeled as a continuous variable using 3-year average volume) and outcomes were assessed using the Spearman rank correlation coefficient. Correlation analyses were conducted for the overall 3-year period and separately for each calendar year (2022, 2023, and 2024) to evaluate temporal trends. Statistical significance was defined as a 2-sided *P* value <.05. All analyses were performed using SAS version 9.4 (SAS Institute).

This study used deidentified administrative data and was deemed exempt from Institutional Review Board approval. Consent was not required.

## Results

### Hospital Trends by Year

Between 2022 and 2024, 118 academic hospitals performed 10,394 transcarotid TAVR procedures across 354 hospital-year observations (Table 1). Total encounters per hospital rose progressively from 3086 in 2022 to 3543 in 2023 and 3765 in 2024 (Table 2). Per-hospital average encounters also showed a modest upward trend over 3 years from 26.2 to 31.9 encounters, although the increase was not statistically significant (*P* = .085). LOS, ICU utilization, CMI, and mortality remained stable from 2022 to 2024 (*P* > .05 for all).

**Table 1:** Hospital-level outcomes and process measures by encounter volume quartile

Variable	3-year encounter volume quartile					
	Overall (N = 354)	15-47 (N = 87)	48-77 (N = 87)	78-111 (N = 90)	112-312 (N = 90)	*P* value
Encounters by quartile, n	2618.8	972	1826	2871	4725	
LOS observed days, mean (SD)	6.0 (3.3)	7.5 (5.1)	6.0 (2.7)	5.5 (2.3)	4.9 (1.8)	**<.0001**
LOS expected days, mean (SD)	5.0 (2.1)	6.1 (2.9)	5.1 (1.9)	4.8 (1.5)	4.2 (1.3)	**<.0001**
LOS index, mean (SD)	1.2 (0.4)	1.2 (0.5)	1.2 (0.5)	1.2 (0.4)	1.2 (0.3)	.84
% ICU encounters, mean (SD)	53.3 (30.7)	70.7 (26.0)	54.0 (36.0)	46.9 (24.4)	42.5 (27.8)	**<.0001**
ICU days, mean (SD)	4.2 (3.5)	4.4 (4.5)	4.0 (3.7)	4.1 (3.1)	4.0 (2.2)	.38
Deaths, n (%)						**.084**
0	212 (59.9)	59 (67.8)	52 (59.8)	58 (64.4)	43 (47.8)	
1	104 (29.4)	20 (23.0)	25 (28.7)	22 (24.4)	37 (41.1)	
2	26 (7.3)	7 (8.0)	4 (4.6)	8 (8.9)	7 (7.8)	
3	9 (2.5)	1 (1.1)	4 (4.6)	1 (1.1)	3 (3.3)	
4	2 (0.6)	0 (0.0)	2 (2.3)	0 (0.0)	0 (0.0)	
5	1 (0.3)	0 (0.0)	0 (0.0)	1 (1.1)	0 (0.0)	
% Observed mortality, mean (SD)	2.4 (4.3)	4.0 (6.7)	2.7 (4.2)	1.6 (2.6)	1.3 (1.6)	.67
% Expected mortality, mean (SD)	2.6 (2.7)	3.7 (4.3)	2.6 (2.2)	2.2 (1.7)	1.8 (1.1)	**.009**
Mortality index, mean (SD)	0.9 (1.6)	1.1 (2.2)	1.0 (1.6)	0.6 (1.1)	0.9 (1.4)	.23
% Early death, mean (SD)	0.6 (2.1)	0.9 (3.5)	0.6 (2.0)	0.4 (1.1)	0.3 (0.9)	.91
CMI, mean (SD)	3.0 (0.9)	3.5 (1.3)	3.0 (0.9)	2.9 (0.7)	2.6 (0.5)	**<.0001**
Complication rate (×1000), mean (SD)						
Ischemic stroke	26.2 (23.6)	19.1 (24.7)	30.4 (31.7)	29.8 (19.9)	25.4 (14.6)	.15
Vascular complications	9.4 (13.5)	11.0 (16.5)	11.9 (18.7)	5.9 (7.5)	8.8 (7.3)	.38
Major bleeding	37.3 (35.0)	58.9 (47.7)	35.0 (25.3)	33.1 (32.6)	22.8 (18.5)	**.0006**
MI	5.0 (9.8)	4.7 (12.1)	6.2 (11.5)	4.5 (7.6)	4.5 (7.8)	.45
Heart failure	170.2 (65.4)	169.5 (94.8)	167.9 (43.4)	164.3 (68.0)	178.8 (45.0)	.62
Pericardial effusion and tamponade	6.4 (14.9)	7.2 (17.2)	9.8 (22.6)	3.5 (6.0)	5.2 (7.6)	.44
Pacemaker implantation	4.6 (8.2)	2.0 (7.4)	7.0 (9.6)	2.6 (6.9)	6.6 (7.7)	**.001**
TIA	18.0 (16.9)	15.7 (24.7)	21.9 (17.2)	16.6 (12.2)	17.7 (10.6)	.17
AKI	6.3 (13.7)	6.9 (20.6)	8.6 (12.7)	6.0 (11.9)	3.6 (6.0)	.30

Hospital-level encounter volume, outcomes, and process measures aggregated over the study period and stratified by hospital encounter volume quartile. Volume quartiles were defined by the cumulative number of encounters over 3 years. LOS observed days represent postprocedure hospital days, LOS expected days are risk-adjusted expected postprocedure days, and the LOS index is the observed-to-expected ratio. % ICU encounters denote encounters requiring ICU care, and ICU days represent ICU days per encounter. Observed and expected mortality reflect in-hospital mortality rates, with the mortality index defined as the observed-to-expected ratio. Early death refers to in-hospital death early during the index hospitalization. Complication rates are reported per 1000 encounters within each volume quartile and include ischemic stroke, vascular complications, major bleeding, MI, heart failure, pericardial effusion or tamponade, pacemaker implantation, TIA, and AKI. Continuous variables are presented as mean (SD); categorical variables, as number (%). *P* values compare volume quartiles using the Kruskal-Wallis test for continuous variables and the χ^2^ test for categorical variables. Bolded values indicate statistical significance. *SD*, Standard deviation; *LOS*, length of stay; *ICU*, intensive care unit; *CMI*, Case Mix Index; *MI*, myocardial infarction; *TIA*, transient ischemic attack; *AKI*, acute kidney injury.

**Table 2:** Hospital-level outcomes and process measures by year

Variable	Overall (N = 354)	2022 (N = 118)	2023 (N = 118)	2024 (N = 118)	*P*-value
Total encounters, n	3464.7	3086	3543	3765	
Encounters per hospital, mean (SD)	29.4 (18.6)	26.2 (16.2)	30.0 (18.2)	31.9 (20.8)	**.085**
LOS observed, d, mean (SD)	6.0 (3.3)	6.0 (3.4)	6.0 (2.9)	5.9 (3.6)	.66
LOS expected, d, mean (SD)	5.0 (2.1)	5.0 (2.4)	5.1 (2.1)	5.1 (1.9)	.37
LOS index, mean (SD)	1.2 (0.4)	1.2 (0.5)	1.2 (0.4)	1.1 (0.4)	.23
% ICU encounters, mean (SD)	53.3 (30.7)	52.0 (31.4)	53.3 (30.1)	54.8 (30.8)	.75
ICU days, mean (SD)	4.2 (3.5)	4.2 (4.0)	4.3 (3.6)	3.9 (2.7)	.76
Deaths, n (%)					.91
0	212 (59.9)	73 (61.9)	69 (58.5)	70 (59.3)	
1	104 (29.4)	32 (27.1)	35 (29.7)	37 (31.4)	
2	26 (7.3)	10 (8.5)	9 (7.6)	7 (5.9)	
3	9 (2.5)	3 (2.5)	4 (3.4)	2 (1.7)	
4	2 (0.6)	0 (0.0)	1 (0.8)	1 (0.8)	
5	1 (0.3)	0 (0.0)	0 (0.0)	1 (0.8)	
% Observed mortality, mean (SD)	2.4 (4.3)	2.5 (4.3)	2.3 (4.2)	2.4 (4.5)	.99
% Expected mortality, mean (SD)	2.6 (2.7)	2.3 (2.1)	2.5 (2.6)	2.9 (3.2)	.12
Mortality index, mean (SD)	0.9 (1.6)	1.1 (2.0)	0.8 (1.3)	0.9 (1.6)	.99
% Early death, mean (SD)	0.6 (2.1)	0.5 (1.7)	0.8 (2.7)	0.4 (1.8)	.42
CMI, mean (SD)	3.0 (0.9)	2.9 (0.9)	3.0 (1.0)	3.0 (0.9)	.54

Hospital-level encounter volume, outcomes, and process measures across the study period, stratified by calendar year. LOS observed days represent postprocedure hospital days, LOS expected days are risk-adjusted expected postprocedure days, and the LOS index is the observed-to-expected ratio. % ICU encounters denote encounters requiring ICU care, and ICU days represent ICU days per encounter. Observed and expected mortality reflect in-hospital mortality rates, with the mortality index defined as the observed-to-expected ratio. Early death refers to in-hospital death early during the index hospitalization. Continuous variables are presented as mean (SD); categorical variables, as number (%). *P* values compare study years using the Kruskal-Wallis test for continuous variables and the χ^2^ test for categorical variables. Bolded values indicate statistical significance. *SD*, Standard deviation; *LOS*, length of stay; *ICU*, intensive care unit; *CMI*, Case Mix Index.

### Hospital and Patient Characteristics

Compared to low-volume centers (Q1), high-volume centers (Q4) treated older patients (median age, 71 years vs 69 years; *P* < .0001), a greater proportion of White patients (85.4% vs 64.5%; *P* < .0001), fewer Black patients (5.7% vs 15.6%; *P* < .0001), and more Medicare beneficiaries (72.4% vs 62.8%; *P* < .0001) (Table 3).

**Table 3:** Patient demographics by encounter volume quartile

Variable	Overall (N = 10,402)	15-47 (N = 924)	48-77 (N = 1880)	78-111 (N = 2873)	112-312 (N = 4725)	*P* value
Age, y, mean (SD)	69.5 (11.2)	66.5 (14.0)	68.9 (11.9)	69.4 (10.9)	70.4 (10.2)	**<.0001**
Age group, n (%)						**<.0001**
<65 y	2823 (27.1)	322 (34.8)	561 (29.8)	768 (26.7)	1172 (24.8)	
≥65 y	7579 (72.9)	602 (65.2)	1319 (70.2)	2105 (73.3)	3553 (75.2)	
Sex, n (%)						**.026**
Female	4006 (38.5)	364 (39.4)	712 (37.9)	1048 (36.5)	1882 (39.8)	
Male	6393 (61.5)	559 (60.6)	1168 (62.1)	1825 (63.5)	2841 (60.2)	
Race, n (%)						**<.0001**
Asian	257 (2.5)	31 (3.4)	83 (4.4)	74 (2.6)	69 (1.5)	
Black	940 (9.0)	144 (15.6)	200 (10.6)	326 (11.3)	270 (5.7)	
Missing	233 (2.2)	39 (4.2)	36 (1.9)	64 (2.2)	94 (2.0)	
Other/Hispanic	663 (6.4)	114 (12.3)	140 (7.4)	154 (5.4)	255 (5.4)	
White	8309 (79.9)	596 (64.5)	1421 (75.6)	2255 (78.5)	4037 (85.4)	
Payer segment, n (%)						**<.0001**
Auto insurance	8 (0.1)	3 (0.3)	1 (0.1)	2 (0.1)	2 (0.0)	
Charity	5 (0.0)	0 (0.0)	2 (0.1)	2 (0.1)	1 (0.0)	
Commercial/private	1865 (17.9)	143 (15.5)	322 (17.1)	532 (18.5)	868 (18.4)	
County medically indigent services	4 (0.0)	2 (0.2)	2 (0.1)	0 (0.0)	0 (0.0)	
Medicaid	788 (7.6)	142 (15.4)	164 (8.7)	212 (7.4)	270 (5.7)	
Medicare	7283 (70.0)	580 (62.8)	1286 (68.4)	1997 (69.5)	3420 (72.4)	
Other	40 (0.4)	8 (0.9)	9 (0.5)	10 (0.3)	13 (0.3)	
Self-pay - cash in full	6 (0.1)	0 (0.0)	0 (0.0)	4 (0.1)	2 (0.0)	
Self-pay – uninsured	129 (1.2)	16 (1.7)	27 (1.4)	37 (1.3)	49 (1.0)	
State-assisted healthcare	54 (0.5)	2 (0.2)	8 (0.4)	14 (0.5)	30 (0.6)	
Unknown	10 (0.1)	4 (0.4)	0 (0.0)	5 (0.2)	1 (0.0)	
Workers Compensation	9 (0.1)	2 (0.2)	3 (0.2)	1 (0.0)	3 (0.1)	

Patient-level demographic characteristics aggregated over the 3-year study period and stratified by hospital encounter volume quartile, defined by cumulative hospital encounters over 3 years. Continuous variables are presented as mean (SD); categorical variables, as number (%). Payer segment reflects the primary expected payer at hospitalization. *P* values compare volume quartiles using the Kruskal-Wallis test for continuous variables and the χ^2^ test for categorical variables. Bolded values indicate statistical significance. *SD*, Standard deviation.

### Patient Complexity and Resource Utilization

**Figure 1:**
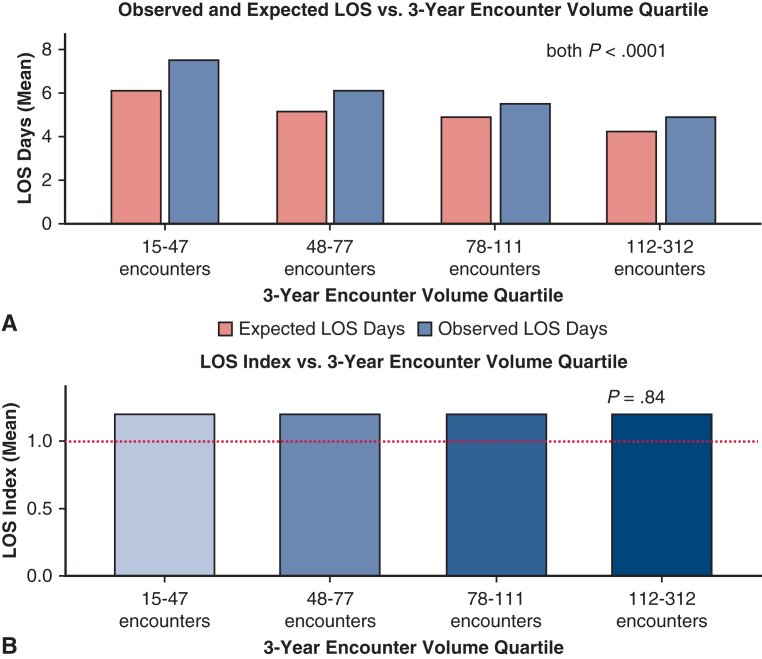
Length of stay (*LOS*) by 3-year encounter volume quartile. A, Bar graph showing mean observed (*blue*) and expected (*red*) LOS in days plotted across 3-year encounter volume quartiles. B, Bar graph showing LOS index (observed/expected LOS) by quartile; a value >1 indicates longer-than-expected LOS, whereas a value <1 indicates shorter-than-expected LOS. The *red dashed line* represents a mortality index of 1.

**Figure 2:**
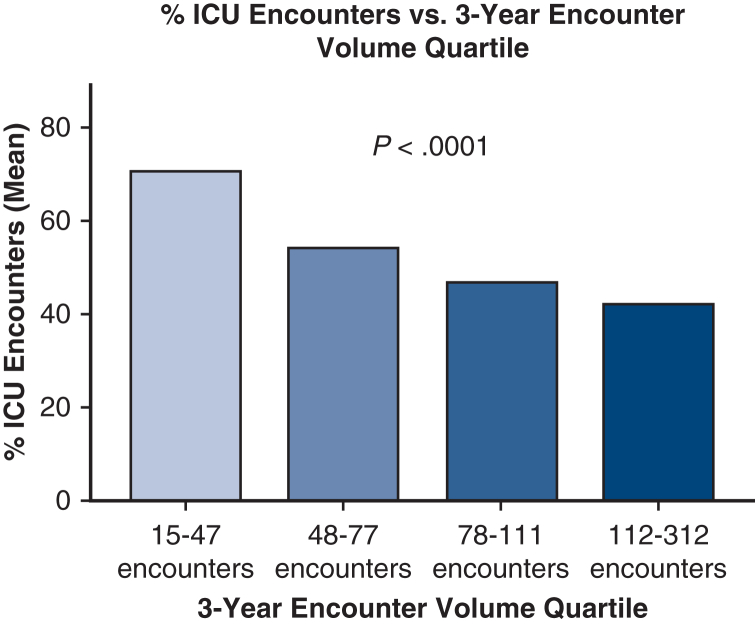
Mean percentage of intensive care unit (*ICU*) encounters by 3-year encounter volume quartile. The bar graph shows the average proportion of encounters classified as ICU-level care across 3-year encounter volume quartiles.

Hospital-level patient complexity decreased with increasing volume, with a median CMI of 3.2 at Q1 centers versus 2.6 at Q4 centers (*P* < .0001) (Table 1). Mean observed LOS declined from 7.5 days in Q1 to 4.9 days in Q4 (*P* < .0001), and mean expected LOS decreased from 6.1 days to 4.2 days (*P* < .0001) (Figure 1). The LOS index remained stable at 1.2 across all quartiles (*P* = .84). ICU utilization decreased from 70.7% in Q1 to 42.5% in Q4 (*P* < .0001) (Figure 2), while mean ICU days remained similar at 3 to 4 days across quartiles (*P* = .38).

### Secondary Complications

Most complications did not differ significantly across quartiles, including ischemic stroke, vascular complications, myocardial infarction, heart failure, pericardial effusion and tamponade, transient ischemic attack, and acute kidney injury (*P* > .05 for all). However, major bleeding and pacemaker implantation rates differed significantly across quartiles, with major bleeding highest in Q1 (58.9 per 1000 encounters; *P* = .0006) and pacemaker implantation highest in Q2 (7.0 per 1000; *P* = .001) (Table 1).

### Mortality Outcomes

**Figure 3:**
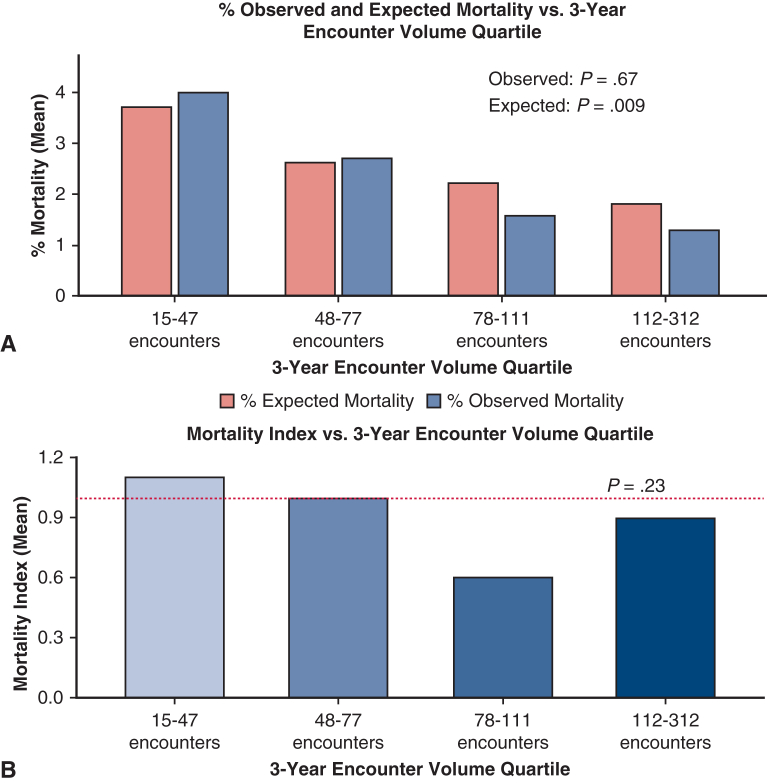
Mortality by 3-year encounter volume quartile. A, Bar graph showing mean observed (*blue*) and expected (*red*) mortality (%) across 3-year encounter volume quartiles. B, Bar graph showing mortality index (observed/expected mortality) by quartile; a value >1 indicates higher-than-expected mortality, whereas a value <1 indicates lower-than-expected mortality. The *red dashed line* represents a mortality index of 1.

**Figure 4:**
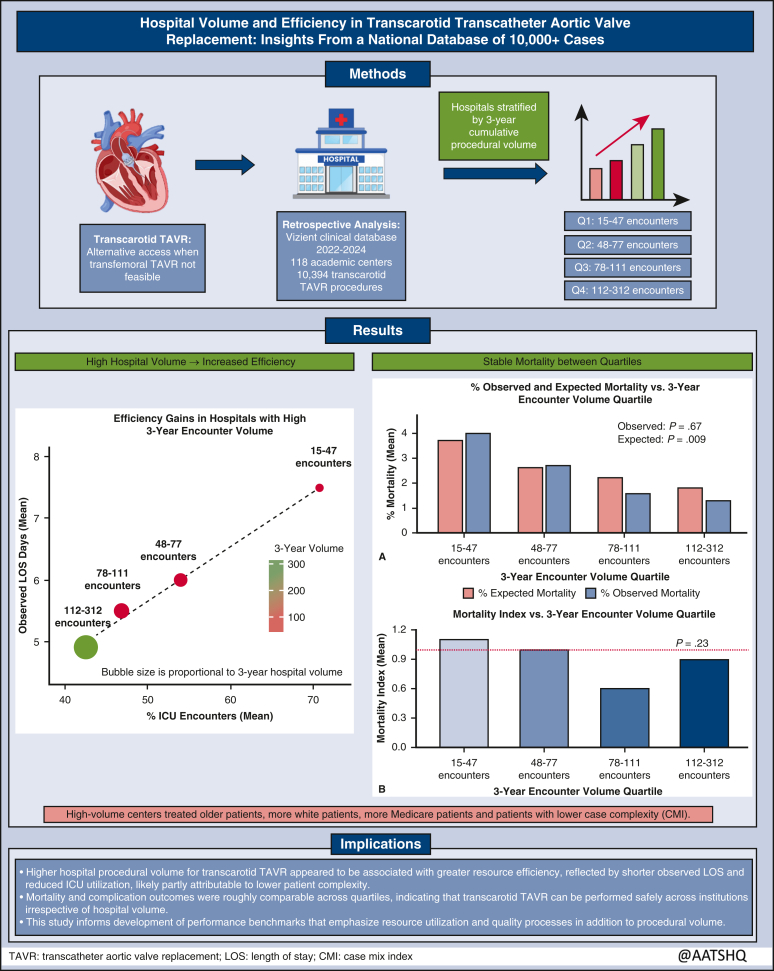
Graphical abstract of the study. Higher hospital procedural volume for transcarotid transcatheter aortic valve replacement was associated with greater resource efficiency, reflected by shorter length of stay and reduced intensive care unit utilization, while mortality remained comparable across volume quartiles. These findings inform performance benchmarking focused on efficiency and care processes rather than on procedural volume alone.

Observed mortality rates ranged from 4.0% in Q1 to 1.3% in Q4, without statistically significant differences across quartiles (*P* = .67) (Table 1, Figures 3 and 4). Expected mortality decreased from 3.7% to 1.8% with increasing hospital volume (*P* = .009). Mortality index ranged from 1.1 in Q1 to 0.9 in Q4 (*P* = .23). Early death rates were low and similar across quartiles (*P* = .91).

### Correlation Analysis

Hospital procedural volume demonstrated weak inverse correlations with observed LOS (r = −0.27), expected LOS (r = −0.25), and CMI (r = −0.25) (*P* < .0001 for all) (Table 4). Volume was not significantly correlated with LOS index (r = −0.06, *P* = .29) or observed mortality (r = 0.04, *P* = .50). Weak correlations were observed with expected mortality (r = −0.13, *P* = .01) and mortality index (r = 0.12, *P* = .03), although effect sizes were small.

**Table 4:** Spearman correlation of hospital encounter volume with outcomes and process measures

Parameter	Encounters			
	Overall (N = 354)	2022 (N = 118)	2023 (N = 118)	2024 (N = 118)
LOS mean observed, d	−0.27 <.0001	−0.16 0.0764	−0.41 <.0001	−0.23 0.0138
LOS mean expected, d	−0.25 <.0001	−0.18 0.0545	−0.36 <.0001	−0.23 0.0114
LOS index	−0.06 0.2918	0.08 0.3662	−0.09 0.3395	−0.13 0.1750
% Observed mortality	0.04 0.5030	−0.04 0.6449	0.15 0.1007	0.01 0.9296
% Expected mortality	−0.13 0.0134	−0.04 0.6645	−0.17 0.0596	−0.22 0.0180
Mortality index	0.12 0.0277	0.02 0.8130	0.27 0.0033	0.086 0.3564
CMI	−0.25 <.0001	−0.21 0.0232	−0.31 0.0006	−0.27 0.0031

Spearman rank correlation coefficients and corresponding *P* values evaluating the association between hospital encounter volume- and hospital-level outcome metrics overall and by study year (2022-2024). All analyses were conducted at the hospital level. *LOS*, Length of stay; *CMI*, Case Mix Index.

Year-specific analyses demonstrated variable correlation patterns (Table 4). In 2023, hospital volume showed stronger inverse correlations with observed LOS (r = −0.41, *P* < .0001), expected LOS (r = −0.36, *P* < .0001), and CMI (r = −0.31, *P* = .0006). Associations between volume and mortality metrics remained weak and inconsistent across all years.

## Discussion

This national analysis of 118 US academic medical centers evaluated the association between hospital procedural volume and outcomes for transcarotid TAVR from 2022 to 2024. Over the 3-year study period, there was a 22% increase in total encounters per year with stable LOS, ICU utilization, CMI, and mortality (Table 2). Steady outcomes despite increasing procedural adoption support the durability of transcarotid TAVR as an alternative access strategy, with minor year-to-year differences likely reflecting local program growth, staffing, or practice changes (Table 4).

Higher institutional volume was associated with greater resource efficiency, reflected by shorter observed LOS from 7.5 days to 4.9 days (*P* < .0001) (Figure 1) and decreased ICU utilization from 70.7% to 42.5% (*P* < .0001) (Figure 2) comparing the lowest-volume and highest-volume centers. The observed inverse relationship between hospital volume and LOS is consistent with established volume–outcome frameworks in cardiac surgery and structural heart interventions, prioritizing perioperative workflows and multidisciplinary team-based care.^9^, ^10^, ^11^

These efficiency gains from a resource-use perspective highlight institutional familiarity with transcarotid access, standardized care pathways, and enhanced coordination among healthcare providers. Prior transfemoral studies have shown similar results, emphasizing that standardized protocol-driven care with minimalist approaches contribute meaningfully to enhancing efficiency without compromising safety.^12^^,^^13^

The LOS index remained stable across quartiles, indicating that shorter LOS at high-volume centers was proportional to expected LOS and was medically appropriate rather than driven by premature discharge (*P* = .84) (Figure 1). Likewise, ICU utilization decreased substantially with increasing volume, while mean ICU days remained similar at 3 to 4 days across quartiles (*P* = .38) (Table 1). This finding suggests more selective ICU triage rather than abbreviated critical care.

Mortality outcomes remained stable across hospital volume quartiles, despite differences in efficiency metrics. Observed mortality, early death, and mortality index did not differ significantly (Table 1, Figure 3). Furthermore, correlations between volume and mortality were weak and inconsistent across years (Table 4). In addition, complications broadly did not differ significantly across quartiles, except for major bleeding and pacemaker implantation, which tended to occur at lower-volume institutions (Table 1). These findings suggest that no clear volume–mortality gradient was observed within this database and study sample, contrasting with volume–mortality relationships reported for surgical aortic valve replacement and previous TAVR cohorts.^14^^,^^15^ However, specific complications may vary by center characteristics or experience, further highlighting the importance of rigorous patient selection and procedural planning. Candidates are typically referred after careful multidisciplinary evaluation and often represent patients unsuitable for transfemoral access but eligible for TAVR. This procedure is also frequently concentrated in academic centers with expertise in complex structural interventions and ongoing training, potentially narrowing drastic differences in outcomes across institutions and learning curve effects.

It is also important to consider that higher-volume centers treated patients with lower predicted risk, as evidenced by the decrease in expected mortality from 3.7% to 1.8% with increasing hospital volume (*P* = .009) (Figure 3). Hospital-level patient complexity, as measured by the CMI, also decreased with increasing volume (*P* < .0001) and was weakly but consistently inversely correlated with procedural volume (Tables 1 and 4). This pattern suggests potential referral or case selection effects in which higher-volume centers may preferentially perform transcarotid TAVR in patients with fewer comorbidities or more favorable anatomy, while lower-volume centers may limit alternative access to higher-risk cases. High-volume centers also treated older patients (median, 71 years vs 69 years), a greater proportion of White patients (85.4% vs 64.5%), and more Medicare beneficiaries (72.4% vs 62.8%) (*P* < .0001) (Table 3). This finding aligns with prior observations in TAVR, where procedural volume is often connected to referral networks, institutional reputation, and patient socioeconomic factors.^16^, ^17^, ^18^ Ensuring equitable access to transcarotid TAVR across populations and geographic regions is essential to mitigate disparities stemming from social determinants of health.

This study has several limitations inherent to retrospective analyses of administrative databases. Outcomes were limited to in-hospital metrics without longitudinal follow-up. The dataset used in this study also provides aggregated institutional summaries rather than fully linkable patient-level observations, which limits the ability to perform detailed patient-level risk adjustment or hierarchical modeling. Additionally, CMI might not fully capture procedural risk, and unmeasured confounding from unaccounted hospital characteristics may persist. Finally, findings from academic centers might not be generalizable to nonacademic or international settings.

In this contemporary national cohort of academic medical centers, higher hospital procedural volume for transcarotid TAVR was associated with greater resource efficiency, while mortality and complications remained broadly similar across institutions. These findings suggest that the procedure can be performed without major differences in outcomes with varying institutional volume and underscore opportunities to optimize and streamline care pathways. As transcarotid TAVR use expands, establishing clear performance benchmarks for efficiency and standardization will be essential to ensure consistently high-quality and equitable outcomes.

### Webcast

You can watch a Webcast of this AATS meeting presentation by going to: https://www.aats.org/resources/hospital-volume-and-efficiency-12532.

**Figure:**
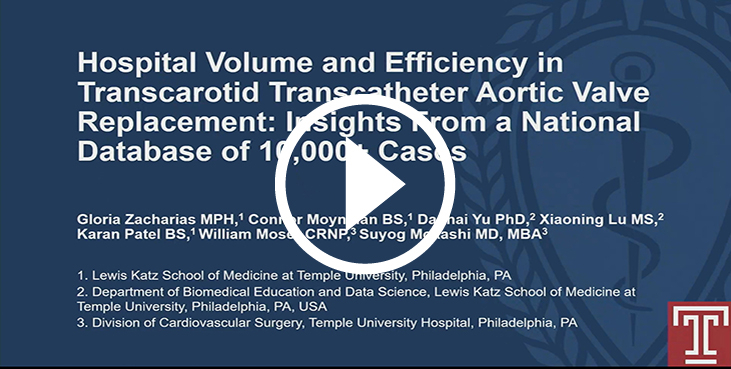

## Conflict of Interest Statement

The authors reported no conflicts of interest.

The *Journal* policy requires editors and reviewers to disclose conflicts of interest and to decline handling or reviewing manuscripts for which they may have a conflict of interest. The editors and reviewers of this article have no conflicts of interest.
